# A Socially Assistive Robot for Stroke Patients: Acceptance, Needs, and Concerns of Patients and Informal Caregivers

**DOI:** 10.3389/fresc.2021.793233

**Published:** 2022-01-25

**Authors:** Ayelet Dembovski, Yael Amitai, Shelly Levy-Tzedek

**Affiliations:** ^1^Department of Cognitive and Brain Sciences, Ben-Gurion University of the Negev, Beer-Sheva, Israel; ^2^Department of Physiology and Cell Biology, Ben-Gurion University of the Negev, Beer-Sheva, Israel; ^3^Zlotowski Center for Neuroscience, Ben-Gurion University of the Negev, Beer-Sheva, Israel; ^4^Department of Physical Therapy, Faculty of Health Sciences, Recanati School for Community Health Professions, Ben-Gurion University of the Negev, Beer-Sheva, Israel; ^5^Freiburg Institute for Advanced Studies (FRIAS), University of Freiburg, Freiburg im Breisgau, Germany

**Keywords:** socially assistive robots, stroke, rehabilitation, focus groups, informal caregivers, patients, participatory design, co-design

## Abstract

Stroke patients often contend with long-term physical challenges that require treatment and support from both formal and informal caregivers. Socially Assistive Robots (SARs) can assist patients in their physical rehabilitation process and relieve some of the burden on the informal caregivers, such as spouses and family members. We collected and analyzed information from 23 participants (11 stroke patients and 12 informal caregivers) who participated in a total of six focus-group discussions. The participants responded to questions regarding using a SAR to promote physical exercises during the rehabilitation process: (a) the advantages and disadvantages of doing so; (b) specific needs that they wish a SAR would address; (c) patient-specific adaptations they would propose to include; and (d) concerns they had regarding the use of such technology in stroke rehabilitation. We found that the majority of the participants in both groups were interested in experiencing the use of a SAR for rehabilitation, in the clinic and at home. Both groups noted the advantage of having the constant presence of a motivating entity with whom they can practice their rehabilitative exercises. The patients noted how such a device can assist *formal* caregivers in managing their workload, while the informal caregivers indicated that such a system could ease *their own* workload and sense of burden. The main disadvantages that participants noted related to the robot not possessing human abilities, such as the ability to hold a conversation, to physically guide the patient's movements, and to express or understand emotions. We anticipate that the data collected in this study—input from the patients and their family members, including the similarities and differences between their points of view—will aid in improving the development of SARs for rehabilitation, so that they can better suit people who have had a stroke, and meet their individual needs.

## Introduction

Nearly 795,000 strokes occur in the United States each year; on average, that means a stroke every 40 seconds (1). It is estimated that by 2030, approximately 3.4 million American adults over 50 will have suffered a stroke (2). With the increase in morbidity rates, the demand for professional, comprehensive, and intensive rehabilitative care tailored specifically to the patient and their injury will also increases (3–10).

Providing the necessary comprehensive care each patient needs can be challenging. Two thirds of stroke patients experience various deficits 6 months after the cessation of their rehabilitation process, and over 50% of them will still have significant disabilities relating to gross and fine motor ability, speech, perception, and cognition, affecting their daily lives and emotional state when evaluated 18 months after stroke (11–14).

When a patient is discharged from a hospital or a rehabilitation center, the balance of care abruptly switches from the formal professional arena to the informal-caregiving arena. Most often, this means a spouse, an adult child, or a friend taking on the burden of care (15, 16). The typical informal caregiver in the US is a 49.4-year-old woman who voluntarily assists a relative for 4.5 years for about 24 hours per week (17, 18). In 2017, it was estimated that family members provide 34 billion hours of treatment per year with an economic value of about $470 billion (16).

**Figure 1:**
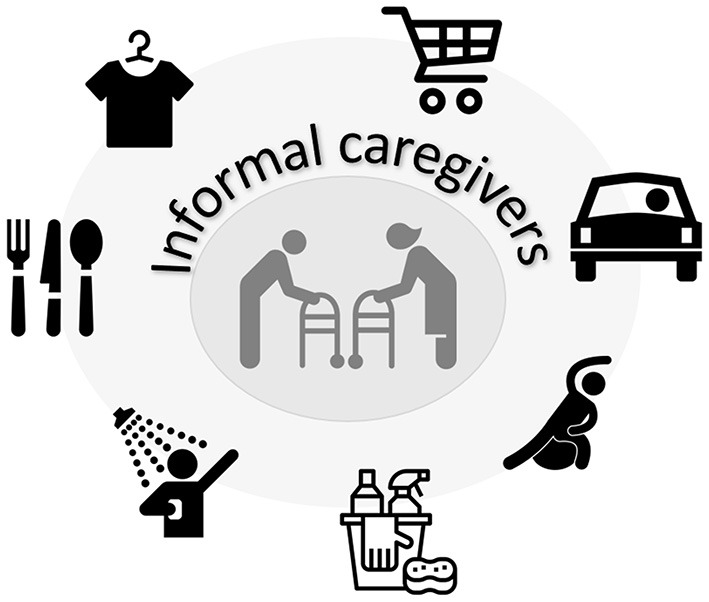
Informal caregivers' roles. Some of roles undertaken by informal caregivers are depicted here, including help with feeding, dressing, traveling, shopping, cleaning, maintaining personal hygiene, and exercising. The breadth of functions many of them fill in the lives of the patients suggest that any improvement in patient independence has the potential to help alleviate some of the burden undertaken by the informal caregiver.

The most common needs of a stroke patient relate to daily activities such as bathing, dressing, and transportation, and less common needs relate to toileting and feeding (19) (see Figure 1). One of the main roles of family caregivers is providing transportation, with nearly 40% of informal caregivers reporting that they accompany patients to routine medical visits (20). The support that informal caregivers provide to patients allows individuals post-stroke to remain in their homes and communities for longer, thus postponing or even preventing institutionalization (20–22). Additionally, informal caregiving helps prevent or delay functional deterioration, reduces the use of medical services, and reduces expenses (20, 23, 24). There is no doubt that support given by informal caregivers is an integral factor in the healing processes of individuals post-stroke.

Lack of such support can have serious consequences: patients who receive inadequate assistance with Activities of Daily Living (ADLs) and Instrumental Activities of Daily Living (IADLs) have been reported to require more physician visits, emergency room visits, and hospitalizations, and to suffer more often from depression (19, 25). Not attending medical appointments or being unable to obtain medical supplies may compromise the medical management of chronic health conditions, underlining the importance of the informal caregiver's role in transportation (19).

Alongside the clear benefits to the patients, this assistance can take a toll on the caregivers' physical and psychological health (26–28). Studies show that lack of caregiver preparation for their role can adversely affect their health, quality of live, and well-being. It has been demonstrated that caregivers face an increased risk of certain medical conditions, such as stroke, depression, fatigue, and more (29–33). It is, therefore, crucial to find ways to support these informal caregivers.

A variety of technological innovations are being developed to assist and ease the burden on professionals, informal caregivers, and patients (18, 34–36). For example, socially-assistive robots (SARs) have been developed to be used in hospitals and in the home, to perform various tasks, such as coaching an exercise session, aiding with ADLs, and encouraging exercise and emotional expression (15, 37–45). The purpose of SARs for rehabilitation is to support and expand independent functioning, reduce the support needed from caregivers, and motivate patients, caregivers, and therapists in coping with the intensive repetitive daily activities required to improve quality of life, health, and psychological well-being (46–50).

It is conceivable that a properly designed interaction with a SAR can offer benefits to both patients and their caregivers in the process of rehabilitation. We posit that it is essential to collect and incorporate these stakeholders' points of view into the process of designing effective interactions with robots for rehabilitation. Within the growing literature on the variety of SARs being developed, few studies explored the similarities and the differences in the needs, expectations and concerns of stroke patients and their informal caregivers, by directly asking the members of these stakeholder groups. For this reason, our goal in the current study was to examine the similarities and the differences in the attitudes, acceptance levels, needs, and concerns of individuals post-stroke and their primary informal caregivers regarding the use of a SAR to promote physical exercise in the rehabilitation process. We aimed to assess these stakeholders' initial reactions to the concept of SARs used in the home and at the clinic, with a focus on a robotic platform which will deliver a combination of cognitive and physical exercises. The current study is intended to complement and serve as a basis for immersive long-term interaction studies in these environments (home and clinic). Specifically, we asked:

(1) How do stroke patients and their informal caregivers perceive the notion of the patients performing rehabilitation exercises, coached by a SAR?
(2) What advantages and disadvantages do they see in such a practice?
(3) What changes or additions should be made to a specific implementation presented to them, so that it better meets patients' needs?
(4) What are their concerns regarding the use of a SAR in the rehabilitation process?

Understanding the needs and differences in opinions among individuals will help to optimize the rehabilitation system for all relevant stakeholders – clinicians [with whom we conducted focus groups in a previous study; see (51)], patients, and their informal caregivers. We expect that the higher the value the patients and their informal caregivers attribute to the SAR, the more likely they are to use it extensively for practicing rehabilitation exercises. Our intention is that the information gathered here will serve researchers, clinicians and engineers when designing interactions with a SAR for healthcare applications.

## Methods

### Research Outline

We used the qualitative method of focus-groups discussions to collect information from patients and their caregivers. The focus groups enable an in-depth discussion that reveals the participants' positions, attitudes, and views regarding various subjects as well as a diversified view of any differences in opinions among the various group members (52–54). The methodology was based on the list of Consolidated Criteria for Reporting Qualitative Research (the COREQ list), which was developed to promote reporting transparency among researchers, while improving qualitative research reliability (55).

### Experimental Protocol

Participants (*N* = 23, age: 68.3 ± 6.8 years; mean ± SD) took part in two experiments: one with individuals who have had a stroke and the other with informal caregivers of stroke patients. We held a total of six focus-group discussions— three with each population group. Participants were recruited using the convenience-sampling approach from Neeman, a nonprofit organization for post-stroke individuals and their families, which works to improve treatment, rehabilitation, and welfare of stroke patients and their families in Israel.

The criteria for inclusion in the study were: stroke patients or informal caregivers over 40 years old, Hebrew speaking. Stroke patients were recruited after the acute stage, if they experienced a rehabilitation process in a hospital, and had a motor impairment in their limbs which limits their movement. Caregivers were recruited if they cared for the individual post stroke three times per week or more. Exclusion criteria for patients were: significant impairment in their comprehension and verbal expression abilities (as assessed by the Neeman group coordinator), additional neurological conditions, undergoing rehabilitation at the time of the study. These criteria were communicated to the Neeman group coordinators, who then invited participants who meet these criteria to the focus-group discussions.

In recruiting participants, we strove to include diverse populations in terms of their geographic residence and socio-economic statuses, and accessibility to large rehabilitation centers.

All meetings were held face-to-face in the location where the Neeman group members usually meet (in Eilat, Hadera and Ofakim), except for the ones located in Haifa which were held using the Zoom video-conferencing software. The moderator had no prior acquaintance with any of the participants, and each participant attended a single focus-group discussion.

Group discussions were held between September 2020 and January 2021. Each discussion lasted between 45–90 min and was videotaped with HC-VX980 Panasonic and DJI OSMO cameras and audiotaped with a ZOOM H1N audio recorder, for further analysis. All discussions were moderated by the first author, a speech-language pathologist, who was a master's student at the time. In addition to the moderator, an assistant from the research team was present in one of the meetings with patients (in Ofakim), and took field notes, and a family-group coordinator was present in one of the meetings with family members (in Eilat); all three are female. At the beginning of each session, the moderator explained the overarching goal of the project: the long-term rehabilitation of stroke patients using a humanoid robot for upper-limb practice; and the specific goal of the focus-group discussions: to get feedback and understand their perceptions regarding SARs for rehabilitation in general, and regarding the specific implementation our research group has developed (15). Our team developed a robot-based gamified exercise platform for long-term post-stroke rehabilitation; the platform uses the humanoid robot Pepper (Softbank Robotics Aldebaran), and includes seven gamified sets of exercises, which are based on functional tasks from the everyday life of the patients, such as reaching to a cup, or turning a key in a lock. Each exercise set comprises a combination of cognitive and physical components. The platform gives the patients instructions, as well as feedback on their performance, and can track their performance over time. Following a brief overview of the platform, and an explanation about the format of the focus-group discussion, participants watched a two-minute video in which healthy participants were seen practicing five different exercise games with the robot. The video shows individuals sitting in front of the robot with a worktable between them. On the table are various everyday objects (e.g., keys, cups) which they are asked to grasp, manipulate and arrange, according to instructions provided by the robot.

The exercise games presented in the video were the Cup Game, the Target Game, the Keys Game, and the Escape Room Game [see (15)]. The video shows the individuals completing the exercise sets and receiving feedback from the robot, having successfully completed the task. The robot's responses included, inter alia: hand clapping, victorious arm gestures, and a jovial waving of the hands. After the participants watched the video, the moderator led a discussion based on 11 questions for the post-stroke patient groups and 12 questions for the family-member groups (see Table 1) which were formulated by the research team, and tested for clarity with members of the extended research group. Participants were asked to describe their personal experiences and note their thoughts and feelings regarding the robotic rehabilitation system, including any perceived advantages and disadvantages, beneficial elements, and what they would have liked to add, upgrade, or change in the system to improve it to suit their needs.

**Table 1:** Discussion questions - Patient groups and Informal-caregiver groups.

**The questions presented in the focus groups**	
**Patient groups**	**Informal-caregiver groups**
**What are your thoughts and feelings regarding the video you just watched?**	
Would you be interested in practicing with Pepper during your rehabilitation process?	Would you be interested in your family member practicing with Pepper during their rehabilitation process?
**What advantages do you think practicing with Pepper has, compared to practicing alone?**	
What disadvantages do you think practicing with Pepper has, compared to practicing alone?	
**What would you add or change for the system to meet your needs?**	**What would you add or change for the system to meet your family member's needs?**
How do you think Pepper could aid and assist you in your rehabilitation?	How do you think Pepper could aid and assist your family member's rehabilitation?
**Do you think there are needs that Pepper cannot address?**	
Do you think there are limitations that could prevent you from practicing with Pepper?	Can you think of any reason why your family member could not practice with Pepper?
**Do you depend on someone else to drive you to your usual treatments?**	**Are you the one driving your family member to their usual treatments?**
If you are arriving independently to treatments, please rate the level of effort you would be willing to invest in traveling for treatments with Pepper.	Please rate the level of effort you would be willing to invest to drive your family member for treatments with Pepper.
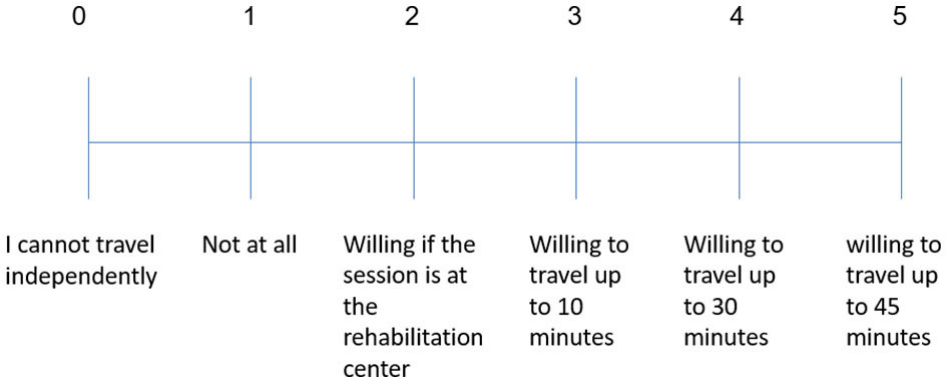	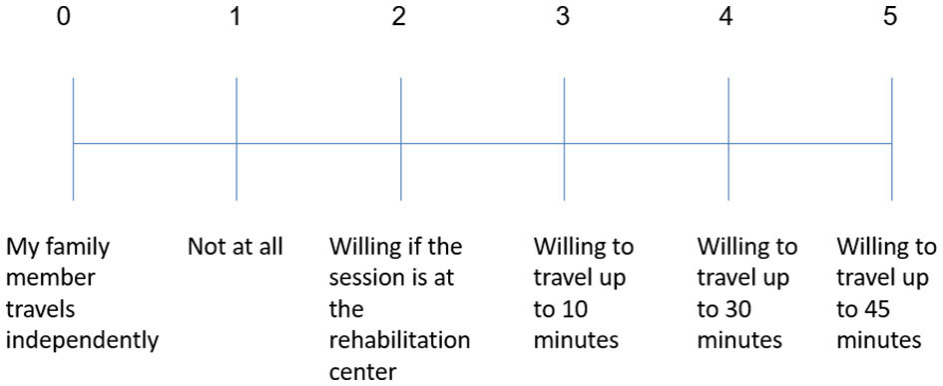
	**Do you think your family member's practicing with Pepper could affect you? If so, in what ways?**
Is there anything you would like to add?	

The video and audio recordings were transcribed and open-coded (applying the inductive approach) by hand using the Framework Method (56). In the thematic analysis process, common themes from the different groups were identified and categorized, as detailed in the Results section below.

After conducting two focus-group sessions, of stroke patients and of informal caregivers (a total of four), it was evident that there was a repetition of the main themes, and data saturation was reached. Therefore, another focus-group discussion was held for each of the two population groups, after which data collection ceased (57). No new variables were noted in the third and final session of each population group. The experimental protocol was approved by the Ben Gurion University of the Negev's ethics committee. All participants gave their written informed consent to participate.

## Results

### Experiment 1—Stroke Patients

In Experiment 1 (*n* = 11, 10 males, 1 female; ages 57-85 years; 69.8 ± 6.7 years [mean ± SD]), the participants were stroke patients (11.2 ± 5.6 years; mean ± SD) 2-20 years post stroke (see Table 2). Three focus groups were held in three different centers: Ofakim (*N* = 3), Eilat (*N* = 3), and Hadera (*N* = 5). The letter in a participant's code in Table 2, as well as in the Results section, indicates the location at which the focus-group session was held.

**Table 2:** Participant demographics—Individuals with stroke (*N* = 11).

**Participant**	**Age (years)**	**Years since the stroke**	**Gender**
O1	57	17	Male
O2	85	5	Male
O3	71	5	Male
E1	69	2	Female
E2	67	10	Male
E3	69	12	Male
H1	66	9	Male
H2	67	16	Male
H3	68	20	Male
H4	78	18	Male
H5	71	10	Male
Average	69.8	11.2	

*The upper-case letter in a participant's code indicates the location of the focus-group session to which they were recruited (O- Ofakim, E- Eilat, H- Hadera)*.

The thematic analysis of the data from the patients' focus groups revealed five main themes: (i) attitudes toward the robot; (ii) motivation for use and feedback; (iii) perceived disadvantages; (iv) adaptability to patients' specific needs; and (v) the use of a SAR as supplementary to standard treatment. Listed below are the details for each of the themes, interlaced with direct quotes from the group discussions; see **Table 4** for a summary of the main issues brought up by the two study populations.

#### Attitudes Toward the Robot

**Figure 2:**
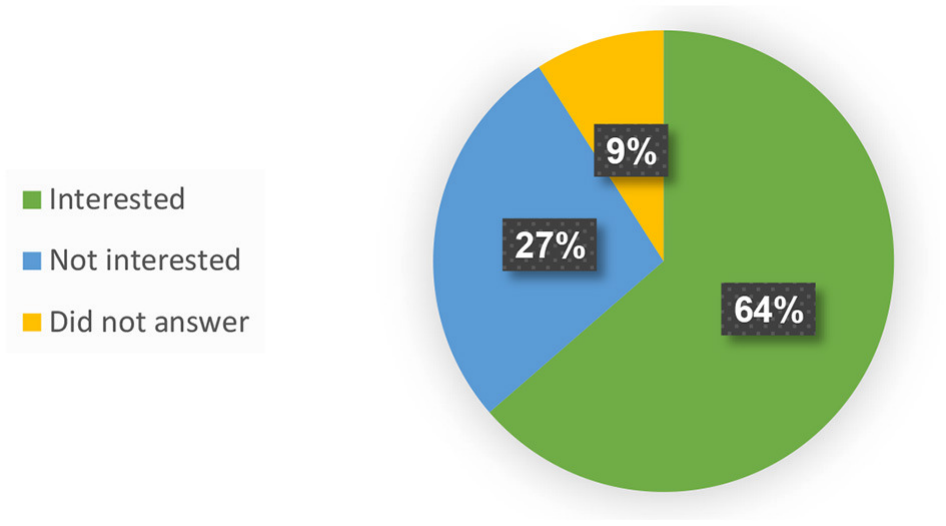
Interest of individuals post-stroke in practicing with a robot as part of the rehabilitation process.

Attitudes toward the robot were mixed. Thirty-six percent of the patients (*N* = 4) stood out for their positive attitude: they thought the robot was interesting, positive, and helpful. Two of those four participants did not see any drawbacks in the system. Thirty-six percent of the patients (*N* = 4) were ambivalent, and 28% of the patients (*N* = 3) opposed the use of the robotic rehabilitation system, saying it lacked the human qualities required in the process of rehabilitation. When they were asked if they would want to use Pepper during their rehabilitation process, most participants (seven, 64%) said they would, three replied they would not, and one abstained (see Figure 2).

#### Motivation for Use and Feedback

Three of the participants noted that discipline and inner motivation were crucial in the process of rehabilitation. Four participants (36%) said the robot could motivate and encourage stroke patients to perform rehabilitation exercises. They indicated that it is exceedingly physically and mentally challenging to do exercises alone and that they often give up as a result, but that they do believe the robot could encourage them to exercise.

“After a stroke, you get occupational therapy [for] 12 treatments. That's it for the whole year … Pepper can help you get more… rehabilitation hours… [and] motivate you even by saying ‘keep going, slowly, slowly… You don't know how much it [the feedback] has an effect, because it's hard [the rehabilitation process]…” (H2)

However, two participants emphasized the difference between the encouragement and support of a robot and the support, and level of feedback that a human can provide.

“There's a huge difference [between a human therapist and a robot assistant]. I prefer people. A person needs feedback” (O2)

See also quote from H2 in the section titled The Human Aspect, below.

Five participants stated that they consider the robot to be an authoritative figure that could supervise them and help them commit to the process. Participant H3, however, did not agree, claiming that the robot could not offer the kind of support that a human therapist can:

“…[the robot] doesn't touch you… it can't really *move* your hands… it took me a long time to put on socks or zip up my pants… It was all done with the help of a therapist…”

Four participants saw the advantages of doing exercises with the robot and also mentioned that it may serve to assist the professionals who already take care of them.

“This thing can take the load off of many people, especially [off the] physical therapists” (O1)

Two of the 11 patients noted that their motivation for treatments in general, and with Pepper in particular, would depend on the support of their family and friends. One mentioned that if their family encouraged them to try this treatment with the robot, they would be happy to do so.

“It also depends on the family… you need the family… as support… The family needs to give you the motivation [to experiment with the robot] … That's very important” (H2)

Two of the participants saw the SAR's value in encouraging the patient to do group exercises, as well.

#### Perceived Disadvantages

##### The Human Aspect

Nine of the 11 patients (82%) expressed their dissatisfaction with the robot's lack of humanity. They referred to its inability to provide guidance and direction, real conversation, empathy, and genuine human contact and interaction.

“[When] a human being… instructs [me] to do something and it's hard for me, they try to help me … a robot won't help me, it will only give instructions, tell me if [what I did] was good or not. But a person who can see that I'm struggling with something would …. help me out” (E3)

“It doesn't have emotions. You need feedback. [A] personal connection is better… A good word is sometimes more important than the entire treatment” (H2)

##### Help With Physical Needs

Participants from the patient group reported a lack of balance, constant falling, and an inability to perform routine actions, such as standing, walking, sitting etc., which require physical support and assistance by caregivers. Six of the participants thought that the robot could not provide this kind of support. Two participants stated that, given an opportunity to practice with technology, they would prefer aid technology (technology that is attached to injured body parts and provides electrical stimulation) which they perceive as more effective.

“I need help with my left hand… physically… Pepper wouldn't have helped me [with that]…” (H2)

#### Adaptability to Patient Disability

##### General Adaptability

The participants made suggestions regarding exercising with Pepper. One suggestion was that the robot gives clear vocal instructions at an appropriate volume. Five participants noted the need to adjust Pepper to accommodate to their physical disabilities. They would have wanted the robot to assist them in standing, sitting, opening doors, and more (see Part 3.5). Two participants referred to Pepper's inability to perform a demonstration after giving instructions. They said that watching a video is not enough for them to understand the exercise and that a physical illustration is mandatory in their view.

“[There should be] a demonstration and then a …video so that a person can understand what [Pepper] wants and then it will be easier… It should be mobile, easy to operate…” (H2)

##### Individual Adaptability

The participants suggested specific adjustments per their individual impairments which they would want programmed into the robot. Seven noted that they would like the robot to help practice motor skills, three noted cognitive skills, and four noted communication skills.

“I think if it should help with everything! With speaking, too” (E3)

#### Robots for Supplementary Practice

Seven participants (64%) stressed that exercising with the robot should not replace conventional treatments with a human therapist, but be done in addition to treatments with a human therapist. Three said that until they have successfully learned how to operate the system, another person should be present during their practice. Three participants stressed that someone must be present in order to mediate and help, at least during the first few sessions.

“[If] I got Pepper, I think it would be good for me, but not as a replacement for someone who helps and guides you… You could [practice with Pepper in rehabilitation], but after a person finishes their part. Human first and Pepper later, [and] not at the same time!” (H3)

“It's better to start with a human… You need someone to guide you on the bigger things like walking, or shopping…”

(H1)“… a person is better … but if I were in a situation where I can have the robot or have nothing, I would prefer having the robot” (O3)

### Experiment 2—Informal Caregivers

In Experiment 2 (*N* = 12, 2 males, 10 females), the participants were informal caregivers of individuals after stroke (ages 48–74 years; 67 ± 6.7 years [mean ± SD]) who have been caring for a stroke patient for 11.5 ± 7 years (mean ± SD; see Table 3). Three focus groups of informal caregivers recruited from the Ofakim (*N* = 4), Eilat (*N* = 2), and Haifa centers (*N* = 6), were held via Zoom video chat due to COVID-19 restrictions.

**Table 3:** Participant demographics—Informal caregivers of individuals with stroke (*N* = 12).

**Participant**	**Age (years)**	**Years caring for the individual with stroke**	**Gender**	**Relation**
o1	70	4	Female	Spouse
o2	72	5	Male	Close friend
o3	63	10	Female	Spouse
o4	62	5	Female	Spouse
e1	69	2	Male	Spouse
e2	48	10	Female	Spouse
h1	67	21	Female	Spouse
h2	70	25	Female	Spouse
h3	70	14	Female	Spouse
h4	66	19	Female	Spouse
h5	73	20	Female	Spouse
h6	74	4	Female	Spouse
**Average**	**67**	**11.5**		

*The lower-case letter in the participants' code indicates the location of the focus-group session to which they were recruited (o- Ofakim, e- Eilat, h- Haifa)*.

The thematic analysis of the data from the informal caregivers' focus groups (the family-member groups) revealed four main themes, which correspond with the main themes from the patient groups: (i) attitudes toward the robot; (ii) motivation for use and feedback; (iii) perceived disadvantages; (iv) adaptability to patient needs; and (v) the use of a SAR as supplementary to standard treatment; see Table 4 for a summary of the main issues brought up by the two study populations.

**Table 4:** The main similarities and differences between the two population groups.

**Issues that came up in the focus-group discussions**	
**Patients**	**Informal caregivers**
**View the robot-based system as an innovative, interesting, and intriguing technology that can motivate the patients to commit to the rehabilitation process**	
Perceive the added value of the system to be: a way of helping to reduce the load from their *formal* caregivers	Perceive the added value of the system to be: it could provide them with more time for self-care and everyday chores, and help prevent friction and disagreements with patients
**Concerned that practice with a robot will** ***replace*** **the care given by formal caregivers; See the value in the robotic system, but refuse to accept it as a** ***substitute*** **for the standard care**	
Mainly want this new technology to assist them with motor needs, primarily with physical support (e.g., balancing, getting up from a chair)	Believe that the gamified exercise system could inspire them and give them ideas for further practice to better facilitate their family member's rehabilitation process
**Think the system should be adapted to the specific needs and capabilities of stroke patients, e.g.,: ease of operation; instructions and feedback written in large lettering, and spoken using a loud voice; repetition of instructions; personalization of difficulty levels; practice of communication, cognition, and memory skills**	
Perceive system's disadvantages to be the robot's inability to: physically demonstrate the instructions; identify and respond to nuances in patient's behavior (e.g., indicators of exhaustion, lack of understanding, etc.); converse with the patients	
**Would like physical contact with the robot, as a means to better practice movement (a guiding touch)**	**Would like the robot to provide comforting physical contact, such as a hug, or a reassuring touch**

#### Attitudes Toward the Robot

Compared with the stroke patients, the informal caregivers had a more optimistic attitude toward using the robot in the rehabilitation process, especially the Ofakim focus group. They expressed great confidence in the robotic system and believed it could perform a wide range of activities and provide care for their spouses.

*All* informal caregivers who took part in the study, when asked if they would be interested in their family member exercising with Pepper during the rehabilitation process, said “yes”. It became apparent that they were willing to try any exercise or treatment that might improve the stroke patients' condition or at least keep it from deteriorating.

“I think it's good, … alongside the 12 [treatments] a year he receives, it would [give him] more [practice] time. I think he can do it at home. During COVID-19 we were not [at the rehabilitation center] a lot and it's harder for him to walk, and [when he exercised] on the bicycle – he said that it was harder than before [because there had been a long break from treatments]” (o2)

#### Motivation for Use and Feedback

The robot was perceived as being an interesting, novel, and an innovative new way to do rehabilitation exercises. Most participants (10 out of 12) thought that the robot had a significant advantage over self-practice because it could motivate patients into action, thus promoting progress. One participant noted that they perceived the robot as an authoritative figure that could aid their spouse in committing to and persisting in their practice. One of the participants in the Haifa focus group mentioned that motivation for exercising could also come from group practice with the robot, a statement with which all five of the group members agreed.

Four participants noted that Pepper could reduce costs, since it can be used at any time and for an unlimited number of treatment sessions.

“*… Therapists … cost more money … [The robot] is one more thing [they can use] … in rehabilitation. From what I know, the more you practice the better… [Pepper] can add interest and provide another form of rehabilitation”* (o4)

It seems that the informal caregivers were motivated to have their spouses use the robot not only because it can encourage the performance of exercises that can improve the patients' condition, but also because the system can give them (the family members) ideas for how to practice with the patient. They noted it may also provide them with an indication of their spouses' progress and allow them to have some free time, thus helping to prevent friction and conflict.

“It really gives us ideas on how to carry on… gives us some confidence” (o1)

“I'm skeptical regarding the ability of [a person with] cognitive limitations to understand what the robot wants, its instructions… on the other hand, I think it can spare us, the caregiving spouses, a lot of negative interactions with our partners because … our involvement with treatments created a lot of antagonism, resistance, and … anger. If someone else can do the work, I think it's good because even in the [traditional] treatments, … when we went to occupational therapy, the therapist would give us something to do … and I was the one who had to deal with him and give him instructions. If a robot can do that, it would reduce some of the friction and I see that as a positive thing” (h4)

“It can set us free. We could leave them with Pepper for 40 minutes and come back to see what they had done” (h5)

On the other hand, two of the participants were quite concerned that the stroke patients would not want to use or cooperate with the robot due to fear of or indifference toward technology (both patients were 57 years old when the stroke occurred).

Six of the participants (all of the Haifa group) saw the SAR's potential in motivating the patient to do group exercises, as well. It should be noted that the topic of using the SAR for group exercises was brought up by the participants, and was not in response to a question by the group mediator.

#### Perceived Disadvantages

##### Human Aspects

Seven of the 12 informal caregivers emphasized that the robot was missing the human capacity to understand the patient. They believe that Pepper will miss small gestures that stroke patients use due to speech and communication impairments and might fail to clarify instructions when misunderstandings arise.

“… [the caregiver] need[s] to know how to hug and smile… the fact that it's a robot and not a human … would not [make my spouse] happy to cooperate” (h5)

#### Adaptability to Patient Disability

The issue of adaptability of the robot, which came up both in the patient groups and in the family-member groups, is comprised of two components: general adaptability –features that must be in all rehabilitation robots; and individual adaptability –features that can be customized for the individual's medical needs.

##### General Adaptability

The informal caregivers noted that the stroke patients may suffer from significant cognitive injuries and that their auditory comprehension and ability to understand instructions may be compromised. Four of the participants opined that the robot would not be suitable for their spouses due to cognitive impairments that make it difficult to understand oral instructions. Three participants mentioned that the robot must provide physical demonstrations of the required activities in order for the patients to understand their instructions; one mentioned that written instructions should be in a large font; and three others mentioned that the robot must have a relatively long waiting time for a response from the patient.

##### Individual Adaptability

Specific adjustments need to be made in the robot's system to suit each stroke patient's particular difficulties. The most common adjustments mentioned were intended to accommodate impairments in communication, writing, reading, retrieval, and memory. Three of the informal caregivers would have liked for the robot to help practice motor skills; five mentioned cognitive skills; four mentioned communication skills; and four participants mentioned social contexts (reading a book or a newspaper to the patient), as well as activities of daily living (such as getting dressed).

“It is hard for my husband to read quickly, and he needs big lettering… [H]e can't hear well, so we got him headphones to raise the volume. There are so many [types of] injuries… [E]ach is different… [T]he treatment should be individually tailored” (o4)

One of the participants noted that she would have liked a SAR to help her husband in re-training his emotional-communication skills. She gave the example of reminding him to smile, since he is often in a bad mood, and she hopes that smiling will make him feel better:

“Sometimes he is in such a bad mood, I … say to him ‘Hello, smile;… it's healthy for the body, to smile.' (o1)

Five of the informal caregivers stated that it was difficult for them to assess the robotic system based on the video presented at the beginning of the focus group discussion.

#### Robots as Supplementary Practice

Eight of the informal caregivers emphasized that the robot should be used to supplement, rather than replace, the work of clinicians. Three of them stressed that someone must accompany it to mediate and help, at least in the first few sessions.

“[The robot should only be] extra help… only if it is in addition to the [standard] treatments… [I]f it's just the robot, then it's not relevant… after the [standard] treatments are done and then there's an option for [practicing with] a robot. I think that could happen” (e2)

#### Informal Caregivers' Extent of Effort

**Figure 3:**
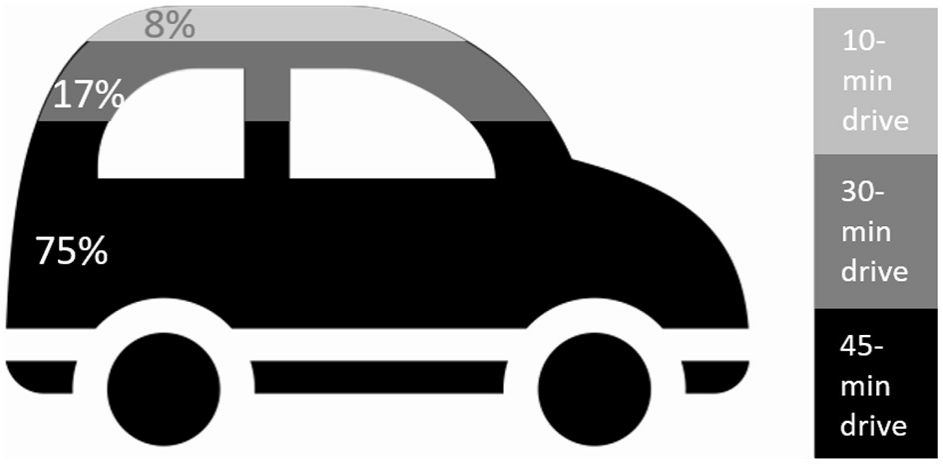
Willingness of informal caregivers to drive the patients specifically for training sessions with the robot. One hundred percent of the participants in the informal caregivers' groups drive their family members to errands and treatments. One hundred percentage of them indicated that they will be willing to drive them specifically for training sessions with the robot, with 92% of them willing to drive 30–45 min for that purpose.

All the family members who participated in the focus groups were the informal caregivers who did most of the driving of stroke patients to their various treatments. We asked the family members how long they would be willing to drive the patients to practice with a robot, as a proxy for assessing the extent of effort they were willing to put into getting their family members to sessions with the robot. The possible responses were: “unwilling”, “willing if the treatment [with the robot] is at the treatment center” (i.e., the rehabilitation center where all other treatments are given), and “willing to travel up to 30/45 minutes” (to a location separate from where the standard treatments take place). The results showed that 100% of the informal caregivers were willing to drive the patients for practice sessions with the robot, with 92% of those willing to drive between 30–45 mins for that purpose (the maximum driving time we asked about was 45 min; see Figure 3). Those who said that they would prefer to travel <45 min mentioned that they do not like or are unable to travel long distances. One mentioned that she would prefer first to check whether her spouse would even want to cooperate with this practice, and only then would she be willing to make the necessary effort.

## Discussion

The aim of the research was to study and analyze the attitudes of stroke patients and their informal caregivers toward the use of SARs during the rehabilitation process. We therefore conducted two sets of focus groups—one comprised of stroke patients and the other of informal caregivers (family members)— to explore the projected levels of use and acceptance, and to understand the two groups' needs and concerns with regards to the rehabilitative system we developed, which presents to the participants a combination of cognitive and physical challenges (15, 51).

The analysis of the data we collected revealed a number of parameters that impact the projected acceptance and use of the robot in the rehabilitation process. These parameters can be divided into two categories: those that relate to the stroke patients and their informal caregivers, and those that relate to the robot.

### Parameters Relating to the Individual

#### Attitudes Toward the Robot

Almost two-thirds (64%) of the participants in the patient groups expressed their desire to exercise with the robot, and those who were reluctant to do so, explained that it was because the robot lacked human qualities and could not meet their physical needs. The attitude expressed by the majority corresponds with results we reported from a user questionnaire (USEQ) administered to 10 patients who had the robot-based rehabilitative system we developed over a five-to-seven-week period, for a total of 15 sessions each; they indicated their wish to continue training with the platform with a score of 4.3 ± 1.0 (out of 5; 15). In a series of in-depth interviews we conducted with nine of those patients, they indicated that their motivation to continue using the system was primarily affected by the perceived functional benefit^1^; this finding was echoed by a strong correlation we found between participants' evaluation of the contribution of the system to their rehabilitation and their willingness to keep training with it (15): “…it is not the mere use of technology that increases the motivation of the person to practice, but rather it is the appreciation of the technology's effectiveness and its perceived contribution to the rehabilitation process”. The minority attitude in the current study reflects the other side of this spectrum: a lack of belief in the functional benefit that training with the robot would bring, leads them to express disinterest in the training.

All participants in the family-member groups expressed interest in having the patient do exercises with the robot, indicating their belief that *any* practice may serve to advance the patients' medical condition. The stroke patients, on the other hand, focused on the functionality of the treatment tool, what it can or cannot do, and formulated their opinion based on this factor. It is important to understand this attitude that functionality dictates use, as it will ultimately impact whether patients will try to use the system. Indeed, it has been previously shown that users that view the robot positively will want to use it and will do so often (58, 59).

#### Motivation and Feedback

Both stroke patients and their informal caregivers view the system as an innovative, interesting, and intriguing technology that can motivate the patients to commit to the rehabilitation process. Only the stroke patients saw added value in the system in so far as it could reduce the workload for their formal caregivers. Indeed, studies have shown that SARs and assistive technologies can ease the load of both formal and informal caregivers (35, 60, 61). Frennert et al. (62) specifically showed that robots were perceived as beneficial to the working conditions of formal caregivers, as a resource for decreasing health costs, and as a way to increase the quality of treatment, seeing as robots can work around the clock without sleeping or being distracted by personal matters.

As for the informal caregivers, the motivation for using the robot was twofold: (1) the positive contribution of the training toward their spouse's rehabilitation; and (2) the benefit of using the robot to them (the family members). They indicated that this gamified exercise system could inspire them and give them ideas for further practice to better facilitate their family member's rehabilitation process. Furthermore, they believed that training with the robot could provide them with more time for self-care or everyday chores and might even reduce friction and disagreements.

Indeed, the notion that the social robot can alleviate some of the burden—be it physical or emotional—from informal caregivers came up in a study by Moharana et al. (63), who designed assistive robots for collaboration with informal caregivers of patients with dementia. One of the caregivers who participated in this study wished that the robot would “take the role of a bad guy” by telling the patient they have to stop eating unhealthy food, thus preventing an argument between the spouses.

These insights provided by the informal caregivers—on how a robot may help patients not only directly, but also indirectly, by helping their caregivers, and easing some of the potential tensions that may develop between them during a long rehabilitation process—highlight the importance of including the informal caregivers in the process of designing assistive technology for the benefit of patients.

#### Robots as a Supplementary Practice

The participants were worried that practicing with a robot would replace the human care given by formal caregivers and made it very clear that while they see the value in training with the robot, they refuse to use it as a substitute for standard treatments, but rather in addition to those.

#### Technological Experience

The informal caregivers raised concerns regarding the cooperation of their family members with the robot over time. Their reasons included indifference to technology, difficulty in understanding how to operate the robot, and a lack of trust in technology, which may lead to avoidance (64, 65). It should be noted that these concerns did not arise from the stroke patients. The informal caregivers mentioned that at least during the first few sessions, until patients gain experience, a person must be present in order to mediate and help, before patients practice alone with the robot. The finding that family members were concerned about the technological barrier to using the robot echoes the findings of Frennert et al. (66) which aimed to assess how different users—adults and their informal caregivers—see or envision the potential role of a robot in their lives. They found that all the informal caregivers thought the relatives in their care (their adult parents) could not learn how to use and operate the robot. It may be that this concern is misplaced: in our long-term study with patients, they responded to the question “Was the information provided by the system clear to you?” with a score of 4.9 out of 5, and to the question “Were you able to control the system?” with a score of 4.8 out of 5 (15).

### Parameters Relating to the Robot

#### Adaptability

Previous studies have indicated that for a robot to meet the needs of the individual, it should be user-friendly, safe, reliable, with a human voice, and moderate movement (66–70). These desired characteristics were echoed by participants in the current study, who indicated that they would have liked for the robot to have the following attributes and functions: mobility; ease of operation; instructions written in large lettering; a loud voice and repetition of instructions with suitable intonation to emphasize parts of the sentence; adjustments to different levels of difficulty; capacity for longer response times by the user; and practice for language and communication impairments (speech, reading, and writing), cognition, memory, social contexts (like reading books) and other motor impairments specific to their injuries (such as hemispatial neglect). This expectation for a multi-modal assistive device echoes studies that show that a combined approach improves function—e.g., in upper-limb function (71) and in gait parameters (72) post-stroke.

Our research group previously assessed the opinions and recommendations of expert clinicians regarding the rehabilitation platform that developed with the Pepper robot for people recovering from a stroke (68). Both the participants of the current study and the expert clinicians (formal caregivers) from the Feingold Polak et al. study (68) mentioned the topics of flexibility and an encouraging reward system. The experts stated that the robot should be customizable to patients' unique needs and conditions; one of the ways to do this, they suggested, is to assure that instructions are spoken clearly and slowly and to have the robot say/express kind words of encouragement (68). While the participants in the current study did not mention anything regarding the response times of the robot, the clinicians in Feingold Polak et al. (68) indicated that the robot should respond quickly. Indeed, patients who used this robotic platform over a 5–7 week period noted that they wish it would react as quickly as a human would (15); they also noted their desire that the robotic platform would be tailored to their individual needs.

One of the main perceived disadvantages of the robot in the current study was its inability to perform a physical demonstration of the instructions to patients, and its inability to notice small nuances; to “read” the patient in certain situations, such as lack of understanding, exhaustion, reluctance to continue exercising, etc. Stroke patients noted they want the technology to also assist them with motor needs: physical support, balancing, and lifting.

In addition, the stroke patients in the current study indicated their need for physical contact. The issue of contact was also mentioned by the informal caregivers, but unlike the stroke patients, who mentioned they needed the physical contact for movement guidance, the informal caregivers referred to the human ability to encourage and support. The informal caregivers used a hug as an example of a significant and useful human expression. Indeed, studies with SARs like “KASPAR” and “Paro” have suggested that humans seek some physical contact when interacting with a robot in social situations (73) and that physical contact with a robot can improve mood and relieve physical pain (74). It is possible that the participants in the current study expected human-like qualities (physical assistance, touch, interaction, and expression of emotion) from this technological platform (75), as they are yet unfamiliar with non-human social platforms which may assist in other ways in the process of rehabilitation.

#### Human Aspects

Both population groups stressed the lack of human traits in the robot; some perceived it as a cold machine, unable to express or understand emotions, converse, or react to a changing situation. They saw it as an automated object with specific, preset answers or instructions that preclude natural conversation and expression of emotion. They mentioned the need to have some sort of social connection with the robot. Interestingly, these issues were not brought up by patients who had trained with the system over a 5–7 week period (15), suggesting that exposure to the benefits the system has – in terms of improving upper-limb function – may change the a-priori perceptions of what the system's characteristics should be. This notwithstanding, it seems it would be advantageous if the system could provide both the training platform and a more human-like connection; Busso et al. (76) suggested that when a system can recognize facial expressions and decipher their meaning, it can assist the user and meet their needs more accurately.

#### Personality

It has been found that a robot with a caring, empathetic, and friendly personality encourages more interaction between it and the individual (77–79). Goetz and Kiesler (80) presented the idea that the robot's personality must match its purpose; the results of their study demonstrate that the participants enjoyed interacting (while engaging in strenuous exercise) with the “playful robot” more than with the “serious, concerned robot”. However, they were less inclined to perform the “playful robot's” requests, and as a result, practiced less with it. These results suggest that users require care and empathy, but also authoritativeness and assertiveness that will motivate them to continue and persevere with their physically straining exercises. It is thus encouraging that both the patients and the informal caregivers in the present study perceived the robot as an authoritative figure that would motivate the users to exercise.

### The Extent of Effort the Informal Caregivers Were Willing to Exert

All the patients in this study rely on their informal caregiver's transportation to arrive at their treatments or doctor appointments, and all of the informal caregivers in this study drive their spouses. Means of transportation and distance from medical centers are considered potential major barriers to the accessibility and utilization of medical care (81). It has been found that those who have an informal caregiving support system that provides them with transportation visit their health care services 1.6 times more than those who do not (81). Distance has been studied mainly in the context of rural areas with low population density versus urban areas and was found to be one of the most influential parameters affecting health outcomes and one of the most significant barriers to health care accessibility (82, 83). The greater the distance from treatment centers is the less frequent are patients' visits (81, 84–86).

In this study we wanted to test whether distance-time is one of the factors that will influence the informal caregivers' decision to drive or not to drive the patients for sessions with the robotic platform. We found that 100% of the informal caregivers were indeed willing to make the effort to drive their family members for treatments with the SAR; 92% of them were willing to drive between 30–45 mins for that purpose (the maximum driving time we asked about was 45 min).

### SAR for At-Home Rehabilitation

We conducted the study in 2020, the year that COVID-19 was declared a global pandemic (87). The informal caregivers indicated that during this period, the standard treatments were canceled or reduced in volume and that patients were left without rehabilitation for an extended period. This disruption to the rehabilitation process may have hampered their recovery. Both stroke patients and informal caregivers saw the added value in having such a guided-exercise system in their own homes, especially during a global pandemic.

### Summary of the Main Differences Between the Responses of the Two Study Populations

While there were similarities between the responses of the participants in the patient groups and in the informal-caregivers groups, there were some notable differences as well, which we summarize below:

1. All (100%) of the informal caregivers were interested in having their family member practice with the SAR, while 64% of the stroke patients expressed their desire to exercise with the robot; those who were reluctant claimed the robot lacked human qualities and would not meet their physical needs.
2. The informal caregivers suggested that any kind of practice could aid the patients and improve their function. The stroke patients, on the other hand, were more cautious and sought to establish the functionality of the treatment tool prior to use.
3. Beyond the perceived benefits to patients, the informal caregivers saw the benefit that using the SAR offered the caregivers themselves (mainly providing them with more free time), while the stroke patients indicated the benefit it offered the *formal* caregivers (mainly reducing their workload).
4. The informal caregivers expressed a concern that indifference to technology, or inability to effectively operate it may lead patients to avoid using it. This concern was not noted by any of the stroke patients.
5. Both groups indicated the importance of physical contact in rehabilitation; The caregivers stressed the importance of emotional touch, and that the robot lacks the ability to touch and hug the patients as a sign of encouragement, while the stroke patients stressed the importance of a guiding touch, and that the robot lacks the ability to indicate to them the correct movement by physically moving their body in the desired way.
6. The participants in the patient groups noted they expected technological tools to provide them physical assistance with standing, sitting, walking etc., and the robot's inability to provide such physical assistance was perceived as a significant disadvantage. This disadvantage was not mentioned by any of the informal caregivers.

### Study Limitations

This study had several limitations. First, the sample size was relatively small; the study was conducted during the COVID-19 pandemic, which limited the ability to gather people together for a discussion session due to lockdowns, cancellations of support group meetings, and social distancing regulations. Some potential participants were reluctant to attend in-person discussion groups due to their at-risk statuses. The total number of participants in our study was 23: 11 stroke patients and 12 informal caregivers. Ideally, future research would include a larger sample size.

Second, there was no equal representation of gender in the focus groups. In the patient groups there was only one woman, and in the family-member groups (the informal caregivers) there were only two men.

Third, it was not possible to mobilize the robot and provide hands-on experience. Instead, the participants were shown a short, two-minute video of the robot doing exercises with healthy individuals. The participants noted it was hard for them to evaluate the robot's abilities and imagine it in action, since they had no first-hand experience with the technology. Notably, despite this limitation, most stroke patients were interested in using a SAR for their rehabilitation. The current study serves to complement the 2-year in-clinic long-term interaction study we conducted with patients using the robotic rehabilitation platform we developed (15), as well as focus-group discussions we held with formal caregivers of individuals post-stroke (51).

Fourth, the level of the stroke patients' physical impairment was not examined on an individual basis; it is, therefore, unknown whether different impairment levels were represented.

Lastly, as in any focus-group discussion, it is possible that not everyone felt comfortable expressing their genuine thoughts and opinions, especially if they differed from the rest of the group members' views. It is possible that if personal interviews had been conducted, different opinions would have emerged.

### Summary and Conclusions

We found that both population groups (patients and family members) had a positive attitude toward using robotic technology in rehabilitation; specifically, a platform that provides a multi-modal intervention, combining cognitive and physical training. They thought that the SAR could motivate, encourage, and help users commit to the long process of rehabilitation, while stressing the importance of personalized adaptation of the robot's behavior to their needs. Participants also noted that they would like the robot to have human-like qualities alongside an authoritative personality to help users comply with their exercise regimen. They thought the robot should be able to react to the individual, provide proper feedback, guide, reflect, demonstrate, converse, and express emotions. As many SARs are designed to help users but do not provide this full range of functionalities, we suggest that it is important to speak openly to patients and their informal caregivers about their specific goals and how they can fit within the range of abilities of the assistive robot.

The participants in the focus groups opined that a SAR would be effective at their rehabilitation facility as well as their home; they expressed that the robot could help its users improve their movement skills and give them additional ideas for activities, freeing up blocks of time for the caregivers, thereby easing their sense of burden. As the “care gap”—the gap between the care that people need and what the healthcare system can offer them (which only widened during the COVID-19 pandemic)—widens, SARs offer patients additional training time in the clinic or at home, and the caregivers generally seemed pleased with the idea (88–91).

This study provided the informal caregivers with a rare platform to express their views, thoughts, and opinions on robotic technology for rehabilitation using qualitative tools, which allowed them to shed some light on and delve deeper into their own experiences. The informal caregivers may be affected mentally, physically, and financially in a process that could go on for years after the event. They hold first-hand knowledge of the injury and the needs of the patient, which is why it is important to collect and analyze information from this population to get a fuller picture of how stroke patients can be treated better and how the caregivers' burdens could be eased.

We hope that this work can serve as a basis for future works developing technological tools for rehabilitation.

### Future Recommendations

Further studies should test which factors have a greater effect on the level of acceptance and use of the robot: factors that derived from the individual as opposed to factors that derive from the robot. It would be interesting to test whether the age of the patients affects their acceptance and use patterns of this technology, and whether these attitudes change over time within a long-term interaction. Finally, it is important to investigate the potential benefits and disadvantages of using a SAR in the home and at the clinic, using a combination of qualitative data collection with objective outcome measures, such as clinical scores.

## Data Availability Statement

The original contributions presented in the study are included in the article. Further data are not publicly available so as not to compromise the privacy of research participants.

## Ethics Statement

The studies involving human participants were reviewed and approved by Ben Gurion University of the Negev's Ethics Committee. The patients/participants provided their written informed consent to participate in this study. Written informed consent was obtained from the individual(s) for the publication of any potentially identifiable images or data included in this article.

## Author Contributions

AD and SL-T designed the experiment. AD collected the patient data, analyzed and interpreted the patient data, and wrote the manuscript. SL-T supervised the experiment and the writing of the manuscript. SL-T and YA secured the funds for the study. YA reviewed and critically commented on the manuscript. All authors read and approved the final manuscript.

## Funding

The research was partially supported by the Helmsley Charitable Trust through the Agricultural, Biological and Cognitive Robotics Initiative, by the Marcus Endowment Fund, and the Paul Ivanier Center for Production Management, all at the Ben-Gurion University of the Negev. Financial support was provided by the Rosetrees Trust, the Borten Family Foundation, the Robert Bergida bequest, and the Consolidated Anti-Aging Foundation. This research was also supported by grant no. 3000017258 from the Chief Scientist Office of the Israeli Ministry of Health, by the National Insurance Institute of Israel, and received funding from the European Union's Horizon 2020 research and innovation programme under the Marie Skłodowska-Curie grant agreement No 754340.

## Conflict of Interest

The authors declare that the research was conducted in the absence of any commercial or financial relationships that could be construed as a potential conflict of interest.

## Publisher's Note

All claims expressed in this article are solely those of the authors and do not necessarily represent those of their affiliated organizations, or those of the publisher, the editors and the reviewers. Any product that may be evaluated in this article, or claim that may be made by its manufacturer, is not guaranteed or endorsed by the publisher.

## Footnotes

^1^Koren Y, Feingold Polak R, Levy-Tzedek S. Extended interviews with stroke patients over a long-term rehabilitation using human-robot or human-computer interactions (unpublished data).
